# Toward an efficient determination of tissue-free water tritium in food

**DOI:** 10.3389/fpubh.2023.1185938

**Published:** 2023-07-10

**Authors:** Hong Ren, Xiaoxiang Ma, Lei Zhou, Peng Wang, Ting Chen, Xiao Zou, Hua Zou, Shunfei Yu, Yi Cao, Zhongjun Lai, Xiaoming Lou, Yiyao Cao

**Affiliations:** ^1^Zhejiang Provincial Center for Disease Control and Prevention, Hangzhou, Zhejiang, China; ^2^School of Public Health, Suzhou Medical College, Soochow University, Suzhou, China; ^3^School of Laboratory Medicine, Hangzhou Medical College, Hangzhou, China

**Keywords:** determination, tissue-free water tritium, microporous membranes, food, Sanmen nuclear power plant

## Abstract

**Introduction:**

Tritium in the environment constitutes a radiological concern because it can become part of the hydrogen pool in environmental and biological reservoirs and thereby expose people to radiation.

**Methods:**

Tissue-free water tritium (TFWT) analysis in food is an important subject of environmental radiation monitoring which plays an important role in the estimation of health risks from environmental tritium exposure. At present, tritium content in food is generally determined by liquid scintillation counter (LSC). To improve the analytical efficiency in tritium determination, we developed a novel method to treat TFWT in food using microporous membranes.

**Results:**

The microporous membrane treatment method developed in this study has the following characteristics: It has a wide range of application and can process TFWT samples with conductivity below 5 μS/cm. Sample loss for the microporous membrane treatment is approximately 5%. The average treatment time is only 5 min, significantly shortened compared with the currently used atmospheric distillation treatment method (1.5 h). The results of the comparison and spike experiment show that the samples prepared by microporous membrane treatment provides equally satisfactory tritium measurement results as classic distillation method.

**Discussion:**

The developed microporous membrane method is simple to operate, efficient, and environmentally friendly, and effectively improves the analysis efficiency of TFWT in food.

## 1. Introduction

Tritium (^3^H or T) is a radioactive isotope of hydrogen with a half-life of 12.3 years and a beta energy range of 0–18.6 keV, with an average beta energy of 5.69 keV (1). Tritium may cause damage, death and chromosomal aberrations in a wide range of cells, such as germ cells and lymphocytes, in living organisms, affects the functions of the nervous and reproductive system, and even cause cancers (2–4). Tritium in biological samples consists of tissue-free water tritium (TFWT) and organically bound tritium (OBT) (5). TFWT is defined as tritium in water that is not bound to tissue molecules. Many methods can remove TFWT from the fresh samples, such as vacuum freeze-drying (6, 7), azeotropic distillation (8), oven-drying (9, 10), low-temperature desorption method (LTDM) (11), and so on, among which vacuum freeze-drying method is the most widely used. After the tissue-free water existing in the sample tissue, cells, and intercellular spaces is separated and collected, the water is purified and the radioactivity of tritium in the sample typically is determined by a low background liquid scintillation counter (LSC) (12).

The use of LSC to measure tritium in biological samples requires thorough treatment to reduce the quenching effect and improve detection efficiency. Traditionally, purification of tissue-free water in biological samples relies on distillation, a method that is time-consuming and can benefit from further improvement and optimization. The operation of membrane separation is known for its simplicity, cost-effectiveness and easy combination with other separation technologies (13), making it attractive for purifying TFWT. Microfiltration with microporous membranes can trap particles such as gravel, silt, clay, algae, and some bacteria in the solution, while a large number of solvents, small molecules and small amounts of macromolecular solutes can pass through the membrane.

In this work, we developed an efficient, simple, and environmentally friendly membrane separation method for the rapid treatment of tritium in tissue-free water from food. The newly developed method was compared with the existing distillation method in terms of recovery, repeatability, impurity removal, and sample preparation time. The developed method was successfully applied to food samples collected around the Sanmen nuclear power plant (SNPP), the first AP1000 nuclear power plant in the world (14). Discharges from SNPP enter the environment mainly through gaseous effluents and liquid effluents, eventually enter the human body through food chains (15). To the best of our knowledge, this work reports the first dataset on TFWT levels in food within the vicinity of SNPP since its operation.

## 2. Materials and methods

### 2.1. Materials and reagents

#### 2.1.1. Experimental reagents

The following reagents were used in the experiments: potassium permanganate (Hangzhou Xiaoshan Chemical Reagent Factory, KMnO_4_), microporous membranes (Material: Polytetrafluoroethylene, hydrophilic property, pore size of 0.22 μm, Shanghai Anpu Experimental Technology Co., Ltd., diameter of 13.00 mm), scintillation cocktail (PerkinElmer, Ultima Gold LLT, Optiphase Hisafe 3, Ultima Gold and Ultima Gold μLLT), Tritium standard solution (Chinese Academy of Metrology, No. DLhd2021-13,611, 1022.00 Bq/g).

#### 2.1.2. Experimental instruments

The following instruments were used in the experiments: moisture analyzer (Shenzhen Fenxi Instrument Manufacturing Co., Ltd., CYS-1.2), vacuum freeze dryer (LABCONCO, 4 L-105°C), complete distillation unit, conductivity meter (Shanghai INESA Scientific Instrument Co., Ltd., DDS-11A), disposable syringe (Shanghai Anpu Experimental Technology Co., Ltd.,10 mL), Inductively Coupled Plasma Optical Emission Spectrometer (Agilent Technologies Inc.,700 series ICP-OES), Liquid Scintillation Counter (ALOKA, LB7).

### 2.2. Samples collection

**Figure 1:**
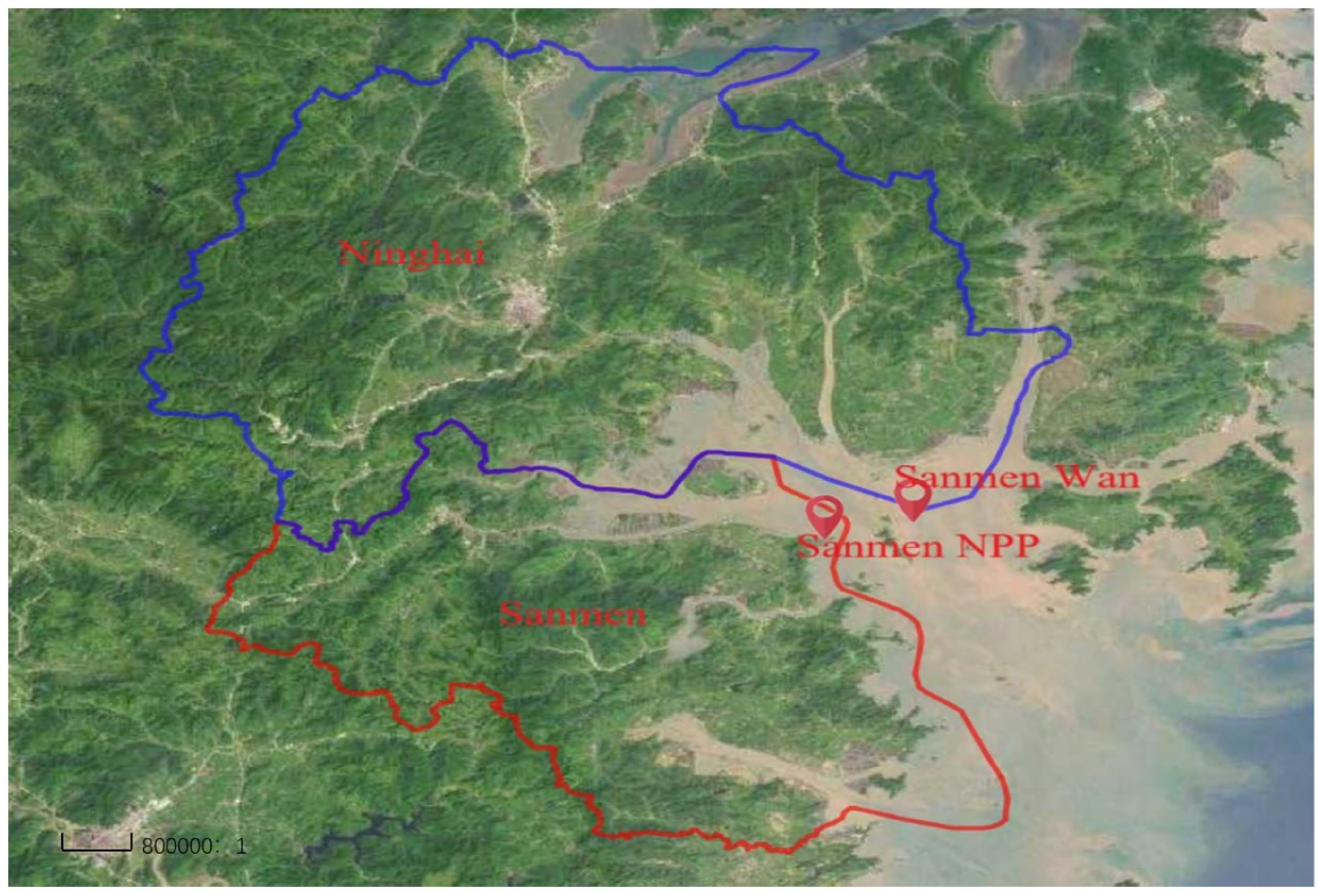
A map showing the location of SNPP and sampling region: Ninghai County and Sanmen County.

Ten types of food were collected around SNPP in April 2022: marine fish, sea shrimps, mussels, seaweeds, sea crabs, cabbage, chicken, celery, potatoes and carrots. The marine fish, shrimps and crabs were washed quickly with purified water after collection, and then dried at room temperature for 10 ~ 15 min. The mussels were washed thoroughly with purified water and decapsulated, and their soft bodies were collected. Seaweeds were collected by removing inedible parts (roots and rotten parts) with a knife, and then washing with purified water to remove the residual sediments. Chicken meat were collected after removing the feather and viscera (16). The sampling quantities were chosen to ensure that the edible part after pretreatment was more than 1 kg; the detailed sampling information is shown in Table 1 and Figure 1.

**Table 1:** Information of sample-collection.

Sample type	Sampling region	Coordinates		Sample weight (kg)
		Latitude (N)	Longitude (E)	
Marine fish	Ninghai County, Zhejiang Province	29°10′42.06″	121°38′32.45″	5
Sea shrimps	Ninghai County, Zhejiang Province	29°10′42.06″	121°38′32.45″	5
Sea crabs	Ninghai County, Zhejiang Province	29°10′42.06″	121°38′32.45″	5
Mussels	Ninghai County, Ningbo City, Zhejiang Province	29°11′03.21″	121°43′31.79″	10
Seaweeds	Ninghai County, Zhejiang Province	29°10′42.06″	121°38′32.45″	5
Cabbage	Sanmen County, Taizhou City, Zhejiang Province.	28°56′49.20″	121°34′0.48″	5
Chicken	Sanmen County, Taizhou City, Zhejiang Province	29°5′2.40″	121°35′48.48″	5
Celery	Sanmen County, Taizhou City, Zhejiang Province	29°5′2.40″	121°35′48.48″	5
Potatoes	Sanmen County, Taizhou City, Zhejiang Province	29°5′2.40″	121°35′48.48″	5
Carrots	Sanmen County, Taizhou City, Zhejiang Province	29°5′2.40″	121°35′48.48″	5

### 2.3. Tissue-free water collection

3 to 8 g of mixed fresh sample was taken in the sample tray of the moisture analyzer, the temperature of the moisture analyzer was set to 105°C, and the mass fraction of tissue-free water in the fresh sample was measured.

One kilogram mixed fresh samples were freeze-dried to obtain tissue-free water.

During the method development, to evaluate the tritium recovery, 100, 500, and 1,000 μL of tritium standard solutions with a radioactivity concentration of 102.2 Bq/g were spiked, respectively in a 250 mL volumetric flask, in which the TFWT obtained after vacuum freeze-drying was added. Tritium solutions with concentrations of 0.04, 0.20, and 0.41 Bq/L, respectively, were thereby obtained and processed following procedures in sections 2.4 and 2.5.

### 2.4. Sample treatment with microporous membranes method

The tissue-free water collected with vacuum freeze-drying was injected into a 10 mL disposable syringe and filtered out of the microporous filter head through a 0.22 μm microporous filter to a beaker. After microfiltration, the conductivity of the processed sample was measured using a conductivity meter. The sample fractions with conductivity less than 5 μS/cm were chosen.

### 2.5. Sample treatment with atmospheric distillation method

To compare with the analytical results based on the membrane method, the collected tissue-free water was in parallel treated with the traditional atmospheric distillation method.

For the distillation, 30 mL of tissue-free water was taken into a 250 mL distillation flask, KMnO_4_ (0.15 g) and a small amount of zeolite were added, the mixture was shaken until physical uniformity was observed. The flask was closed with a glass stopper and connected to the serpentine condenser tube. The temperature during the distillation process was carefully controlled and recorded. After distillation, the conductivity of the condensed water was measured using a conductivity meter.

### 2.6. Measurement of tritium by LSC

Counting efficiency for tritium by LSC is calculated as:


$$\varepsilon =\frac{N_{\chi }-N_{b}}{60\times D}$$

where ε is the counting efficiency of the instrument for tritium (%), $N_{x}$ is the count rate of the standard sample (min^−1^), $N_{b}$ is the count rate of the background sample (min^−1^), D is the radioactivity of tritium added to the standard sample.

To improve the counting efficiency, the scintillation cocktail types and their mixing ratios with the sample were investigated to select the optimal condition. To facilitate the quenching correction, a quenching curve was obtained by adding different amount of quenching agent to a standard sample.

#### 2.6.1. Mixing ratio of sample with scintillation cocktail

The background water and scintillation cocktail (Ultima Gold LLT) were mixed in a 20 mL scintillation vial in the ratio (v/v) of 6: 14, 7:13, 8:12, 9:11, 10:10, and 11:9, respectively and then 100 μL (10.22 Bq/L) of tritium standard solution was added.

#### 2.6.2. Selection of scintillation cocktail

The background water was mixed with a different scintillation cocktail at a time, including Optiphase Hisafe 3, Ultima Gold. Ultima Gold μLLT and Ultima Gold LLT in a 20 mL scintillation vial in the ratio of 8:12 (v/v). Thereafter, 0.1 mL (10.22 Bq/L) of tritium standard solution was added.

#### 2.6.3. Quenching correction

To obtain the quenching curve, a tritium standard solution (102.01 Bq/L) was spiked to a mixture of background water with UltimaGold LLT scintillation cocktail in a 20 mL scintillation vial, different amount of quenching agent CCl_4_ was added according to Table 2. External standard channels ratio (ESCR), a numerical value of the quenching parameter that indicates the quenching level of the sample, was used in this study to correct the samples with different quenching effects.

**Table 2:** Composition of sample solutions for obtaining tritium quenching curve.

Number	Background water (mL)	Tritium standard solution (μL)	UltimaGold LLT scintillation cocktail (mL)	CCl_4_ (μL)
1	7.5	500	12	0
2	7.5	500	12	10
3	7.5	500	12	20
4	7.5	500	12	30
5	7.5	500	12	40
6	7.5	500	12	50
7	7.4	500	12	100
8	7.3	500	12	200
9	7.0	500	12	500

In all cases for tritium measurement in this work, each scintillation vial was tightly closed and mixed vigorously for 1 min, and measured after 12 h of dark adaptation inside the LSC. Each measurement lasted 1,000 min, with the counting channel of 50 ~ 189. For processed samples, unless otherwise specified, the mixing ratio of sample to cocktail was selected as 8 mL:12 mL.

### 2.7. Measurement of metal elements by ICP-OES

Conductivity is related to the concentration of metals contained in the sample. Different metal ions respond differently in ICP-OES. Therefore, several common metal ions are divided into two groups A and B for detection based on their measurement performance including sensitivity, detection limit, wavelength and interferences in ICP-OES measurement.

**Figure 2:**
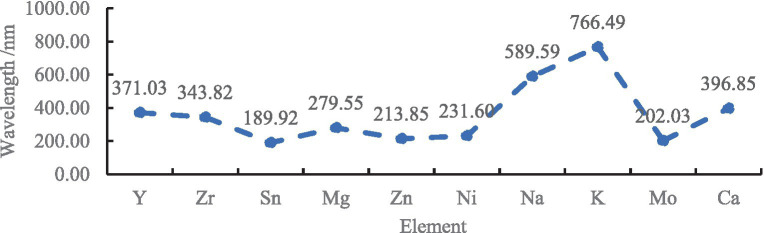
The wavelength of group A metallic ions.

(1) The elements Y, Zr, Sn, Mg, Zn, Ni, Na, K, Mo, and Ca were assigned to group A; standard solutions of each element were added to the untreated TFWT samples and atmospheric distilled TFWTs to prepare solutions with concentrations of 0.0, 2.0, 4.0, 6.0, 8.0 and 10.0 mg/L. Group A samples were detected under the wavelength conditions shown in Figure 2.

(2) Cu, Pb, Cd, V, Ti, Mn, Li, In, Cr, Co, and Sr elements were assigned to group B; standard solutions of each element were added to untreated TFWT samples and atmospheric distilled TFWTs to prepare solutions with concentrations of 0, 0.20, 0.40, 0.60, 0.80 and 1.00 mg/L.

**Figure 3:**
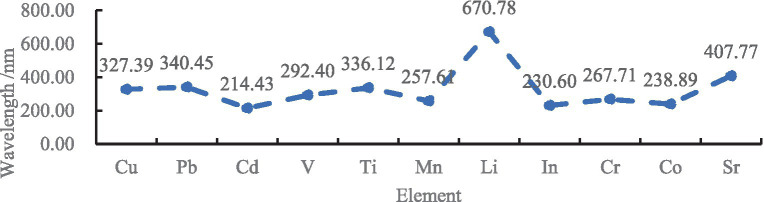
The wavelength of group B metallic ions.

Samples in group B were detected under the wavelength conditions shown in Figure 3.

### 2.8. Experimental flowchart of this study

**Figure 4:**
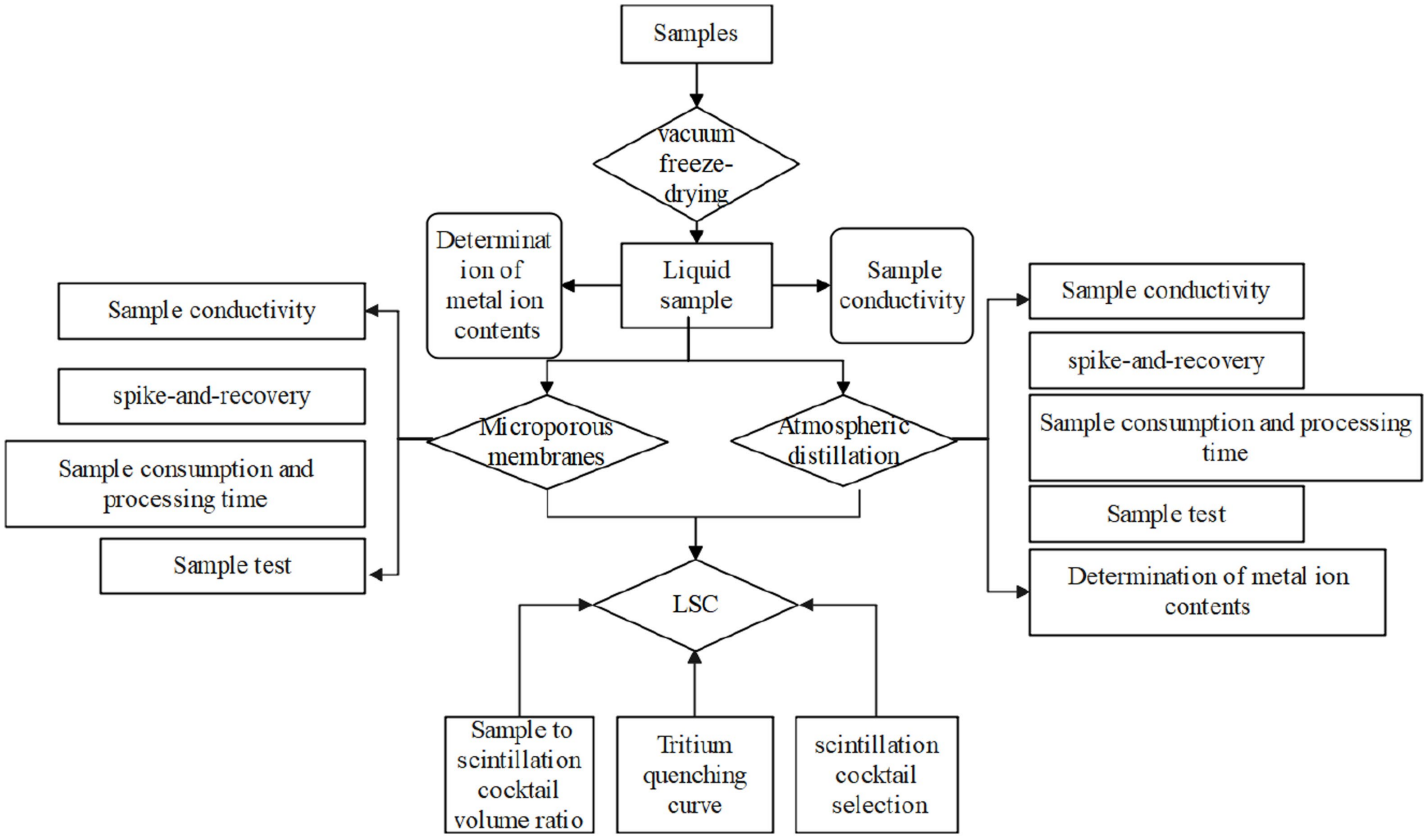
Experimental flowchart.

The entire experimental flowchart of this study is shown in Figure 4. In general, the collected sample was vacuum freeze-dried (2 ~ 4 days) after the measurement of moisture content to collect the tissue-free water. After measuring the conductivity and metal ions concentrations, the tissue-free water samples were splitted and treated by microporous membrane and atmospheric distillation method, respectively. The analytical performance for both methods including the impurity removal (reflected by conductivity and metal ions concentrations), recovery, processing time and analytical accuracy were quantified and compared. The factors (above-mentioned in section 2.5) affecting the detection of LSC were investigated and optimized to improve the counting efficiency.

### 2.9. Calculations and statistics

#### 2.9.1. Lower detection limit

Lower detection limit was according to:


$$LLD=\frac{4.65\;}{0.06\cdot \varepsilon \cdot m}\sqrt{N_{b}/t_{b}}$$

where *LLD* is the lower limit of tritium detection in water (Bq/L), $N_{b}$ is the count rate of the background sample (min^−1^), $t_{b}$ is the measurement time for background specimens (min), ε is the detection efficiency of the instrument for tritium (%), and m is the mass of the water sample taken (g). The lower detection limit of this method is 1.28 Bq/L.

#### 2.9.2. Activity concentration


$$A_{TFWT}=\frac{\left(N_{x}-N_{b}\right)\times \omega \times 1000\;}{60\cdot \varepsilon \cdot m}$$

where $A_{TFWT}$ is the activity concentration of free water tritium in tissues (Bq/kg), $N_{x}$ is the count rate of free water tritium samples (min^−1^), $N_{b}$ is the counting rate of the background sample (min^−1^), ω is the moisture content of food (%), ε and m are as in the previous equation.

For statistical analysis, we used the Mann–Whitney U test and t-test of SPSS 25.0 system. In this study, *α* = 0.05 and *p* < 0.05 was considered a statistically significant difference.

## 3. Results and discussion

### 3.1. Sample applicability

To quantitatively evaluate the applicability of the membrane procedure, the conductivity of water was first evaluated. Conductivity serves as an important indicator for evaluating the presence of salts, ions and impurities in water. Measuring activity concentration of TFWT with the LSC has certain requirements on the conductivity of the sample.

The conductivity of tissue-free water from 10 types of food samples was measured before and after microporous membrane filtration or atmospheric distillation using a conductivity analyzer. The results of all samples showed a conductivity of less than 5 μS/cm before treatment. Since TFWT were obtained at low temperatures (below-50°C), metal ions were not separated. There was no significant change in conductivity after treatment with microporous membranes. The conductivity of some samples decreased after atmospheric distillation, while the conductivity of shrimp, cabbage and chicken increased. The experimental results are shown in Table 3.

**Table 3:** Sample conductivity.

Sample type	Untreated (μS/cm)	Microporous membranes (μS/cm)	Atmospheric distillation (μS/cm)
Marine fish	1.35	1.19	0.25
Sea shrimps	1.24	1.20	2.04
Sea crabs	0.55	0.23	0.49
Seaweeds	0.92	0.89	0.15
Mussels	1.99	1.94	1.06
Cabbage	0.81	0.79	0.84
Chicken	0.19	0.19	0.37
Celery	0.04	0.04	0.15
Potatoes	0.27	0.24	0.24
Carrots	0.06	0.06	0.11

**Figure 5:**
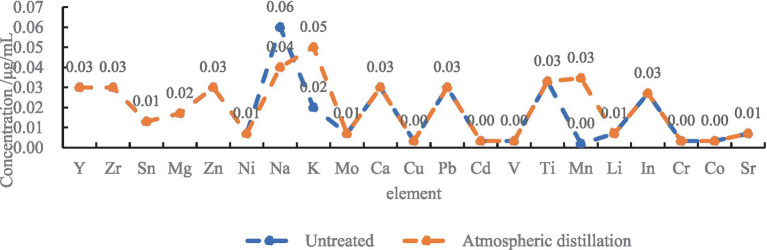
Metal ion content before and after the atmospheric distillation.

The concentration of more than 20 metallic elements in untreated tissue-free water and the water treated after atmospheric distillation were detected using ICP-OES. The 21 ions were detected in the untreated tissue-free water, and the rest of the ions except Na^+^ were below the limit of detection. The K^+^ and Mn^2+^ concentration of atmospheric distilled tissue-free water is higher than those in untreated tissue-free water, possibly due to the evaporation of potassium permanganate into the distillate during distillation treatment. The Na^+^ concentration is lower than that of untreated tissue-free water, which is reduced by distillation treatment (Figure 5).

### 3.2. Sample consumption and processing time

Membrane treatment and atmospheric distillation have great differences in treatment time and sample recovery. The sample treatment by atmospheric distillation takes a long time (close to 1.50 h), and the sample recovery rate ranges from 85.51 to 89.46%, i.e., sample loss of 10.54 to 14.49%. The membrane treatment process is simple, it takes about 5 min, and the sample recovery ranges from 95.16 to 96.50% (sample loss <5%). Comparatively less sample amount is required for microporous membranes treatment. Due to pressure and sample purity issues, in order to prevent cross-contamination, it is recommended to use each membrane for ≤5 times the treatment of the same sample.

### 3.3. Accuracy verification of tritium measurement results

In order to verify the accuracy of tritium measurement results for the microporous membrane treatment method, we compared the experimental results with the traditional atmospheric distillation treatment method. The results are shown in Table 4. The tritium recoveries vary from 88.60 to 118.20% in the membrane method, and 89.60 to 117.16% in the distillation method, respectively. There was no statistically significant difference between the results of distillation and membrane treatment (*p* > 0.05). The relative standard deviation (RSD) using 5 replicates in each batch experiment were less than 5.00% for both methods. The results of *t*-tests between membrane and distillation method were *t* = −1.57, −1.20, and −1.61, with *p* = 0.16, 0.26, and 0.15 at the low, medium, and high levels of tritium spike, respectively.

**Table 4:** Comparison of spiked results.

Spikes level	Serial number	Tritium recovery (%)		*RSD* (%)		*t* ^1^	*p* ^1^	*t* ^2^	*p* ^2^
		Microporous membranes	Atmospheric distillation	Microporous membranes	Atmospheric distillation				
Low	1–1	100.78	100.83	3.37	2.85	−1.57	0.16	0.47	0.66
	1–2	104.70	105.10						
	1–3	103.79	103.14						
	1–4	101.66	104.72						
	1–5	95.95	108.94						
	Average	101.38	104.54						
Medium	2–1	118.20	115.95	1.38	0.54	−1.20	0.26		
	2–2	114.19	115.57						
	2–3	115.06	116.29						
	2–4	114.98	117.16						
	2–5	114.68	116.731						
	Average	115.42	116.34						
High	3–1	91.24	92.31	1.15	1.12	−1.61	0.15		
	3–2	88.60	91.55						
	3–3	90.51	91.56						
	3–4	90.71	89.60						
	3–5	89.65	90.90						
	Average	90.14	91.18						

^1^Spike-and-recovery experience analyze the results.

^2^RSD analyze the results.

The result that the samples treated by both treatment methods for different concentrations of TFWT and both showed high accuracy, with a relative standard deviation from 1.15 to 3.37%. The statistical reveals no significant difference between the results obtained from the microporous membrane and atmospheric distillation method (*p* < 0.05).

### 3.4. Selection of LSC measurement conditions

**Figure 6:**
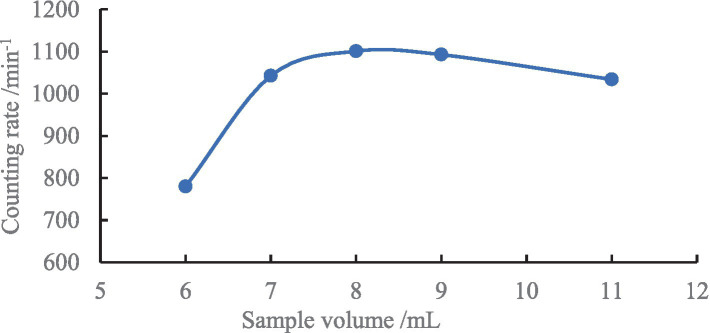
Sample to scintillation volume ratio results.

The mixing ratio of the sample to the scintillation cocktail affects the LSC measurement, as a lower sample volume can result in a lower sample counting rate with higher uncertainty, whereas an excessive sample volume can lead to emulsification thus affecting the detection. Through experimental comparison, it was observed that a volume ratio of 8:12 (sample to scintillation cocktail) provided optimal measurement sensitivity and detection efficiency (Figure 6).

Scintillation cocktails of PerkinElmer with different background count rate, counting efficiency, and anti-quenching ability multiple are commonly used in detection of radionuclides. The results in Table 5 shows that, among the four investigated scintillation cocktails, Ultima Gold LLT exhibits the highest counting efficiency (25.34%) for tritium measurements.

**Table 5:** Scintillation cocktail selection.

Scintillation cocktail type	Counting rate (min^−1^)	Counting efficiency (%)
Optiphase HiSafe 3	141.65	22.97
Ultima Gold	145.64	23.62
Ultima Gold μLLT	153.97	24.98
Ultima Gold LLT	156.19	25.34

### 3.5. Quenching correction

**Figure 7:**
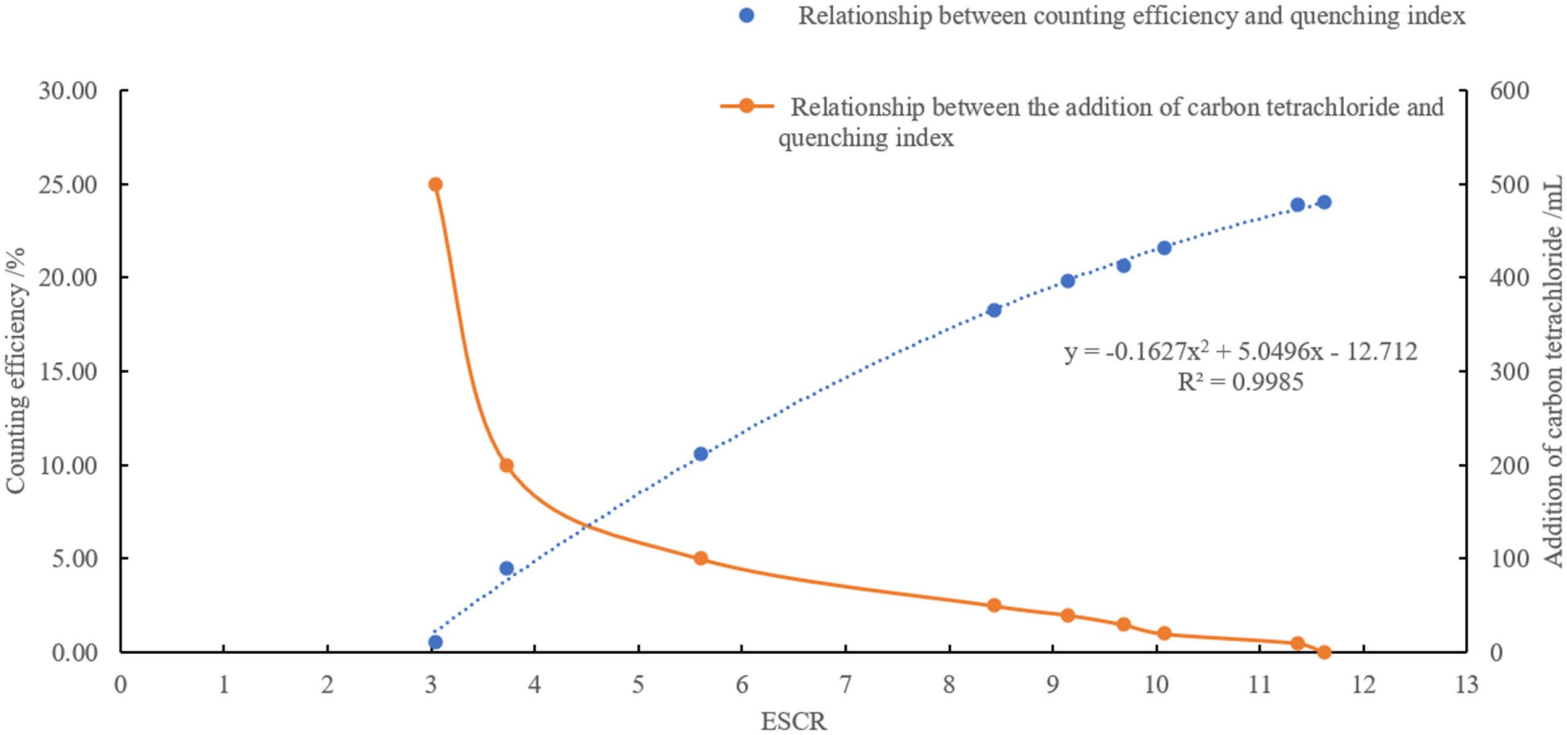
Tritium quenching curve used in this study.

When the activity concentration of tritium is detected by the LSC method, there are quenching effects such as color quenching and chemical quenching, which affect the accuracy of detection. Quenching is unavoidable, and quenching correction is required to make measurements comparable across samples. In this experiment, the external standard method was used to obtain the quenching correction curve. Figure 7 shows that as the quenching agent (CCl_4_) increased from 0 to 500 μL, the ESCR values decreased from 11.62 to 3.04 and the counting efficiency for tritium decreased from 24.05 to 0.57%.

### 3.6. Sample analysis results

Both microporous membrane and atmospheric distillation methods were used to TFWT analysis in 10 food products collected around SNPP. The results are shown in Table 6.

**Table 6:** Liquid scintillation counter-measurement results.

Medium	Activity concentration of tritium in tissue-free water (Bq/L)		Activity concentration of TFWT in the original sample [Bq/kg (fresh)]		*Z*	*P*
	Microporous membranes	Atmospheric distillation	Microporous membranes	Atmospheric distillation		
Marine fish	<1.28	1.35	<0.80	0.85	−0.11	0.91
Sea shrimps	1.95	1.91	1.25	1.22		
Sea crabs	5.49	2.78	3.75	1.99		
Seaweeds	2.67	2.14	1.12	0.90		
Mussels	<1.28	<1.28	<0.91	<0.91		
Cabbage	<1.28	1.38	<1.17	1.27		
Chicken	1.49	2.50	0.95	1.59		
Celery	3.50	4.39	3.06	3.83		
Potatoes	3.66	2.27	3.09	1.91		
Carrots	3.52	1.49	3.17	1.34		

The activity concentration of TFWT obtained by microporous membrane ranged from <1.28 to 5.49 Bq/L and the activity concentration of TFWT ranged from <0.80 to 3.75 Bq/kg (fresh). Whereas under atmospheric distillation the activity concentration of TFWT range found to be <1.28 to 4.39 Bq/L and the activity concentration was in the range of <0.85–3.83 Bq/kg (fresh). There was no statistically significant difference in activity concentration results (*p* > 0.05). The results are shown in Table 6. This TFWT range is similar to that reported by Kim et al. (16, 17) and Baburajan et al. (18), but slightly lower than that measured by Baeza et al. (19) and Baglan et al. (20).

## 4. Conclusion

Based on the above experiments, we conclude that the microporous membrane treatment method developed in this study provides an efficient, fast, simple, and environmentally friendly approach for TFWT analysis. Current experiments have shown good applicability for TFWT samples with conductivities below 5 μS/cm, and whether the method is suitable for samples with conductivity higher than 5 μS/cm will be verified in future studies. Due to pressure and sample purity issues, it is recommended to use each membrane for ≤5 times the treatment of the same sample. Sample loss during the microporous membrane treatment is <5%. The average treatment time is only 5 min, significantly shortened compared with the commonly used atmospheric distillation treatment method (1.5 h). The experiment results demonstrate that the samples prepared through microporous membrane treatment provides equally satisfactory results for tritium compared to those obtained through atmospheric distillation.

## Data availability statement

The original contributions presented in the study are included in the article/supplementary material, further inquiries can be directed to the corresponding author.

## Author contributions

HR, XXM, LZ, PW, TC, XZ, HZ, SFY, YC, ZJL, XML, and YYC: data-processing, writing, analyze, operation, and sample collection. All authors contributed to the article and approved the submitted version.

## Funding

This research was funded by the Zhejiang Provincial Foundation Public Welfare Research Project (No. LGC21H260001), Zhejiang Health Science and Technology Plan (No. 2021KY613, 2022RC120, 2022KY130, 2022KY132, and 2023KY643), and Project of South Zhejiang Institute of Radiation Medicine and Nuclear Technology (No. ZFY-2021-K-003, ZFY-2022-K-001, and ZFY-2022-K-006).

## Conflict of interest

The authors declare that the research was conducted in the absence of any commercial or financial relationships that could be construed as a potential conflict of interest.

## Publisher’s note

All claims expressed in this article are solely those of the authors and do not necessarily represent those of their affiliated organizations, or those of the publisher, the editors and the reviewers. Any product that may be evaluated in this article, or claim that may be made by its manufacturer, is not guaranteed or endorsed by the publisher.
